# Multimodal Assessment of Cerebral Perfusion and EEG Maturation in Preterm Infants at Term-Equivalent Age

**DOI:** 10.3390/children13050647

**Published:** 2026-05-05

**Authors:** Yahui Zhang, Yanxia You, Jianqiu Huang, Yunfeng Liu, Tongyan Han

**Affiliations:** 1 Department of Pediatrics, Peking University Third Hospital, Beijing 100191, China; 2 Department of Ultrasonography, Peking University Third Hospital, Beijing 100191, China

**Keywords:** amplitude-integrated EEG, cerebral maturation, multimodal monitoring, neurodevelopment, ultra-micro angiography

## Abstract

**Highlights:**

**What are the main findings?**

- This study combined ultra-micro angiography (UMA)-based quantitative perfusion studies and amplitude-integrated electroencephalogram (aEEG) functional scores for the first time to reveal the characteristic differences in cerebral microcirculation perfusion in preterm infants of different gestational ages at term-equivalent age (TEA).

**What are the implications of the main findings?**

- By integrating UMA technology and aEEG, this study elucidated cerebral microcirculation perfusion patterns and EEG activity characteristics of preterm infants of different gestational ages, corrected to TEA through multimodal monitoring.
- This investigation of the correlation between microcirculation parameters and EEG, to elucidate the influence of extrauterine development on cerebral circulation–function coupling, potentially facilitates the development of noninvasive monitoring strategies for early intervention in high-risk preterm infants.

**Abstract:**

Background/Objective: Preterm birth poses notable neurodevelopmental risks, with cerebral microcirculatory disturbances potentially contributing to long-term impairment. Existing monitoring modalities lack bedside capacity to evaluate these microvascular changes during critical brain development. We characterized cerebral microperfusion and functional maturation patterns in preterm versus full-term neonates using combined ultra-micro angiography (UMA) and an amplitude-integrated electroencephalogram (aEEG). Methods: In this prospective study, 76 neonates (23 extremely/very preterm [EP/VPT], 27 moderate-late preterm, and 26 term controls) were assessed at term-equivalent age. UMA helped quantify regional microperfusion (color pixel percentage, abbreviated as CPP in this context to differentiate from cerebral perfusion pressure), whereas aEEG (Burdjalov scores) helped evaluate functional maturation at 37–38 and 40 weeks’ postmenstrual age. Results: EP/VPT infants demonstrated significant cerebral hyperperfusion with distinct cortex–white matter perfusion gradients. Although preterm infants showed advanced aEEG maturation at 37–38 weeks, this difference normalized by 40 weeks. Conclusion: Gestation-dependent cerebral hyperperfusion and transient EEG maturation differences in EP/VPT infants at term-equivalent age support the value of UMA–aEEG integration for neurovascular assessment. The observed perfusion–EEG patterns suggest prematurity-specific neuroadaptation, warranting further investigation of long-term functional correlates.

## 1. Introduction

Premature birth, the leading cause of neonatal mortality and neurodevelopmental disorders, constitutes approximately 10% of live births [1]. Late pregnancy is crucial for cerebral angiogenesis, synaptic formation, and myelination; thus, premature infants face dual challenges of unstable cerebral perfusion and unbalanced metabolic demands [2,3,4,5]. Studies have shown that abnormal cerebral hemodynamics in premature infants can occur before structural damage and are closely related to long-term motor and cognitive impairments [6]. Therefore, monitoring and maintenance of early cerebral perfusion in neonates are paramount for brain development and long-term neurological prognosis [7,8,9]. However, most imaging techniques (e.g., arterial spin labeling magnetic resonance imaging [MRI], gadolinium-based MRI, positron emission tomography, computed tomography, and single photon emission computed tomography) are unsuitable for bedside dynamic monitoring of cerebral microcirculation in premature infants owing to limitations such as radiation exposure and the need for sedation [10].

Although functional near-infrared spectroscopy (fNIRS) and traditional Doppler ultrasound can partially assess cerebral blood flow, the former has limited spatial resolution, and the latter can only detect large blood vessels. Neither technique can precisely quantify local microcirculation [11]. Ultra-micro angiography (UMA) technology addresses these limitations by enabling direct visualization of microvascular structures and perfusion dynamics [12,13,14]. Depending on the ultrasound equipment manufacturer, this technology is also referred to as microvascular imaging, superb microvascular imaging, or microvascular flow, among other names.

Meanwhile, the Burdjalov scoring system for amplitude-integrated electroencephalogram (aEEG), an electrophysiological marker of brain functional maturity, has been verified to predict neurodevelopmental outcomes [15]. However, studies on the joint assessment of the association between cerebral microperfusion and electrical activity in preterm infants at corrected term-equivalent age (TEA) using UMA and aEEG are lacking.

Thus, we aimed to integrate UMA quantitative perfusion and aEEG functional scores to reveal the characteristic differences in cerebral microcirculation perfusion in preterm infants of different gestational ages during TEA. We also sought to explore the correlation between microcirculation parameters and EEG to elucidate the influence of extrauterine development on cerebral circulation–function coupling and to provide noninvasive monitoring strategies for early intervention.

## 2. Materials and Methods

### 2.1. Research Design and Ethical Approval

In this prospective, observational, single-center study, we evaluated the changing patterns of cerebral microperfusion and electrical activity in preterm infants from development to full term at corrected gestational age (TEA) and determined the differences among various brain regions.

### 2.2. Research Participants

Inclusion criteria: A total of 182 newborns who were hospitalized in the neonatal intensive care unit and neonatal ward of Peking University Third Hospital from 1 May 2023 to 30 June 2024 were screened; subsequently, 167 newborns were enrolled for the study with informed parental consent.

**Figure 1:**
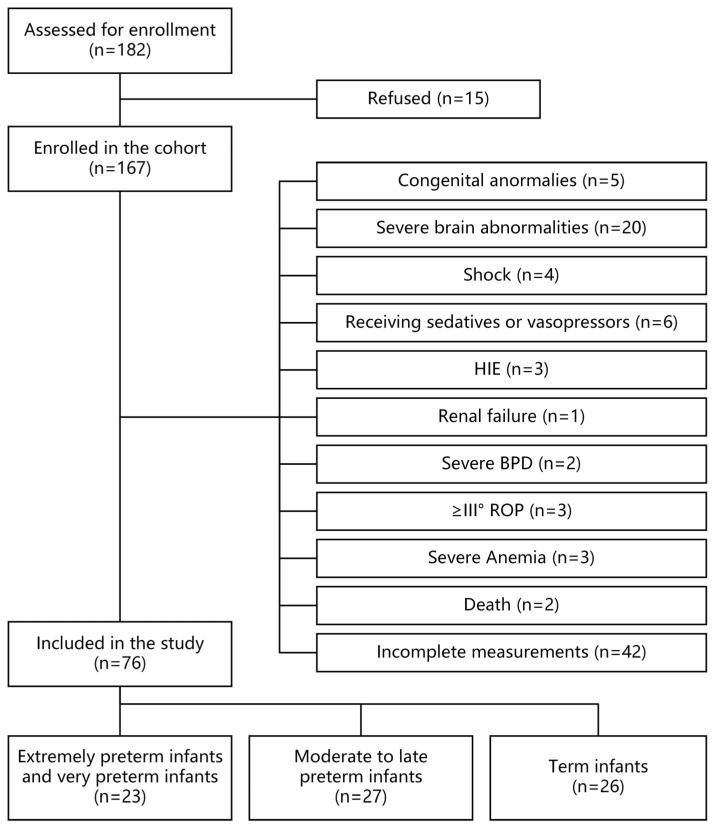
Flowchart of the cohort inclusion in the research study. Hypoxic–ischemic encephalopathy (HIE); retinopathy of prematurity (ROP); bronchopulmonary dysplasia (BPD).

Exclusion criteria: (1) major congenital/chromosomal anomalies; (2) significant neurological pathology (e.g., intraventricular hemorrhage grade > II, stroke, hypoxic–ischemic encephalopathy, white matter damage, periventricular leukomalacia, central nervous system infection); (3) hemodynamic instability (e.g., shock, vasopressor use, hemodynamically significant patent ductus arteriosus [hsPDA]); (4) major organ dysfunction (e.g., respiratory, renal, gastrointestinal, hematologic); (5) respiratory failure or current receipt of high-parameter invasive ventilatory support (mean airway pressure > 8 mmHg), and/or inhaled oxygen concentration > 0.30; (6) sedative use; (7) technical limitations (e.g., sedation, poor acoustic windows); (8) death at 38–41 weeks of correction. hsPDA was previously defined [16], whereas invasive ventilation parameters included mean arterial pressure > 8 mmHg and fraction of inspired oxygen (FiO_2_) >0.30. The screening flowchart is shown in Figure 1.

### 2.3. Instruments and Parameters

Neonatal cranial ultrasound assessments were conducted using a Mindray Resona R9Q system equipped with both C11-3U (3.0–11.0 MHz) and L15-3U (3.0–15.0 MHz) linear array probes for UMA imaging (parameters: 50-dB grayscale gain, 40-dB flow gain, and maintained within safe operating limits [MI 0.4–0.8, TI 0.01]).

### 2.4. Inspection Methods

All infants underwent supine transcranial ultrasound during quiet sleep, with the head in a neutral position and fontanelles exposed. Standard grayscale and Doppler imaging assessed the anterior and middle cerebral arteries (peak systolic velocity [PSV], end-diastolic velocity [EDV], and resistive index [RI], averaged over three cycles). The UMA mode helped visualize parenchymal microvessels and quantify regional perfusion via color pixel percentage (CPP).

### 2.5. Image Acquisition

In accordance with the recommendations of the American Association of Ultrasound in Medicine, the right side of the participant was displayed on the left side of the image during coronal scanning, and the forehead was displayed on the left during sagittal scanning. Image acquisition involved a C11-3U micro-convex probe (Mindray Bio-Medical Electronics, Shenzhen, China) to obtain grayscale sonograms and color Doppler flow imaging (CDFI) of the target area, with the blood flow velocity scale set to a minimum value of 2.9 cm/s. The region of interest (ROI) within the target area was selected; UMA imaging mode was subsequently applied to acquire images and measure the CPP, with the blood flow velocity scale set to a minimum value of 2.5 cm/s.

### 2.6. CPP Measurement

**Figure 2:**
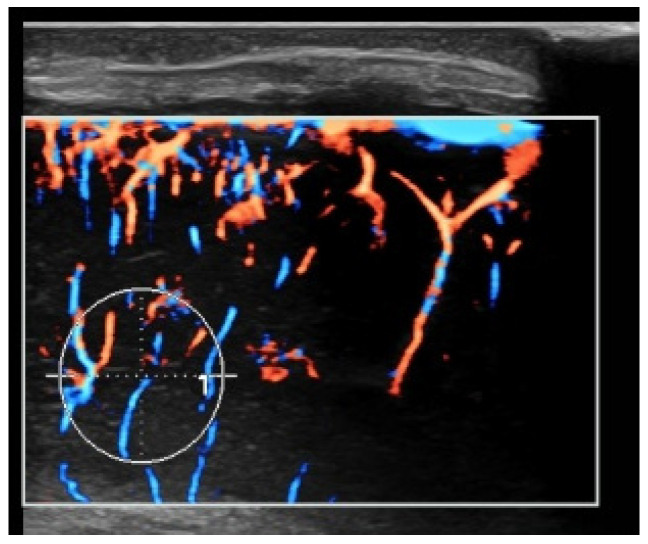
A sampling frame area of 0.79 cm^2^ yielded a color pixel percentage value of 29.29%.

The term CPP in this study specifically refers to color pixel percentage, a microvascular flow metric distinct from cerebral perfusion pressure. Using UMA imaging, we placed 1 cm^2^ ROIs (0.79 cm^2^ area) over five brain regions (frontal/parietal cortex, basal ganglia, midline junction, and white matter) to quantify microvascular blood flow [2]. The system automatically calculated CPP as a flow-richness index. Triplicate measurements per site were averaged to reduce error (Figure 2).

### 2.7. Anterior Cerebral and Middle Cerebral Artery Measurements

CDFI helped visualize the anterior cerebral artery (ACA) and middle cerebral artery (MCA) along with their major branches, whereas pulsed-wave Doppler measured PSV, EDV, and RI to assess neonatal intracranial hemodynamics.

On the day before and after the UMA examination, the enrolled children underwent aEEG examination. All records were analyzed by an aEEG analyst.

Assessments were uniformly conducted during the day (9 am–3 pm) to avoid the influence of circadian rhythms and to cover both quiet and active sleep cycles.

### 2.8. Monitoring Methods

Monitoring using the Cadwell aEEG system (Cadwell Laboratories, Inc., Kennewick, WA, USA), according to the international 10–20 system positioning method, involved placement of recording electrodes at positions C3, P3, C4, and P4 (spacing: 75 mm); reference electrodes were placed at the frontal midline and back/shoulders, 25 mm above the top of the head, and fixed with a special net cap. All patients underwent ≥6-h continuous EEG monitoring; data were analyzed using Cadwell’s proprietary EEG review software (Version-3.1.509). Results were interpreted by blinded, experienced technicians after excluding monitoring data with significant artifacts or impedance > 10 kΩ (Table 1).

### 2.9. Evaluation Criteria and Burdjalov Score

For each record, we selected the most stable and uninterrupted ≥6-h period for analysis. The scoring system proposed by Burdjalov [15] was used to score aEEG.

### 2.10. Other Data Collection

To ensure accuracy and completeness, the following data were obtained from the hospital’s electronic data management system: (1) maternal factors (complications, delivery mode), (2) perinatal indicators (Apgar score, resuscitation needs), (3) neonatal parameters (gestational age, growth percentiles, critical laboratory results), and (4) hemodynamic status (blood pressure, SpO_2_, and respiratory support).

### 2.11. Interobserver Consistency Assessment

Two trained physicians (A and B) independently conducted three neonatal cerebral microvascular flow imaging assessments and calculated their mean values while blinded to each other’s interpretations. Interobserver consistency was assessed; discrepancies were resolved by joint remeasurement and consensus.

### 2.12. Data Analysis

Data analysis was conducted using SPSS (version 26.0; IBM Corporation, Armonk, NY, USA). Normality of data distribution was confirmed using the Shapiro–Wilk test, with normally distributed data expressed as mean ± standard deviation (x ± s). One-way analysis of variance was used for intergroup comparison, and Tukey’s honestly significant difference test for pairwise comparison. Non-normally distributed measurement data are expressed as the median (M) and interquartile range (P25, P75), with the Kruskal–Wallis H test used for intergroup comparison and Bonferroni correction for pairwise comparison. A linear regression model was used to calculate the Pearson correlation coefficient to study the relationship between gestational age at birth and cerebral microperfusion at TEA. Counts are expressed as frequencies and percentages. The chi-square test was used for intergroup comparison, and the Bonferroni correction for pairwise comparison. A *p*-value < 0.05 for intergroup comparisons and a Bonferroni-corrected *p*-value < 0.017 were considered significant.

## 3. Results

### 3.1. Between-Group Comparison of Baseline Characteristics

The 76 infants (51.50% female) included in this study were categorized into three groups based on gestational age at birth: 23 extremely or very preterm infants (<32 weeks), 27 moderate-to-late preterm infants (32–36^+6^ weeks), and 26 full-term infants (≥37 weeks). Comparisons of corrected age, sex distribution, proportion of small-for-gestational age infants, cesarean-section rate, and proportion of in vitro fertilization showed no significant intergroup differences. There were no significant differences among the gestational age groups in terms of maternal pregnancy-associated complications and basic vital signs of infants at the time of examination; however, significant intergroup differences in weight and hemoglobin were detected, with lower levels in the extremely premature and very premature groups (Table 2).

### 3.2. Intergroup Comparison of CPP by Brain Regions and ACA and MCA

**Figure 3:**
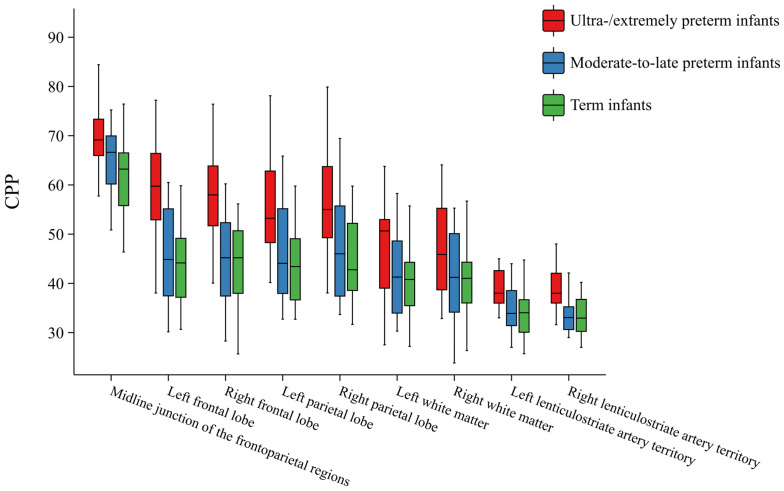
Intergroup comparison of regional color pixel percentage.

The bilateral frontal lobe, parietal lobe, white matter, lenticular artery, and midline CPP differed significantly when grouped by gestational age. In pairwise comparisons, the CPP of the bilateral frontal lobes, parietal lobes, lentostriatal arteries, and midline was highest in extremely preterm and very preterm infants during TEA. Bilateral white matter indicated that the CPP of extremely preterm and very preterm infants during TEA was significantly higher than that in full-term infants, without differences in comparisons with moderate-to-late preterm infants. PSV and EDV of the ACA and MCA showed intergroup differences. Pairwise comparisons showed that the PSVs of ACA and MCA were highest among extremely preterm and very preterm infants and lowest among full-term infants. The EDVs of the ACA and MCA were higher in extremely preterm and very preterm infants than in full-term infants. RI values across all gestational age groups were within the normal range, indicating a normal physiological state and stable great cerebral artery blood flow (Table 3 and Figure 3).

### 3.3. Intergroup Differences in CPP Among Different Regions

**Figure 4:**
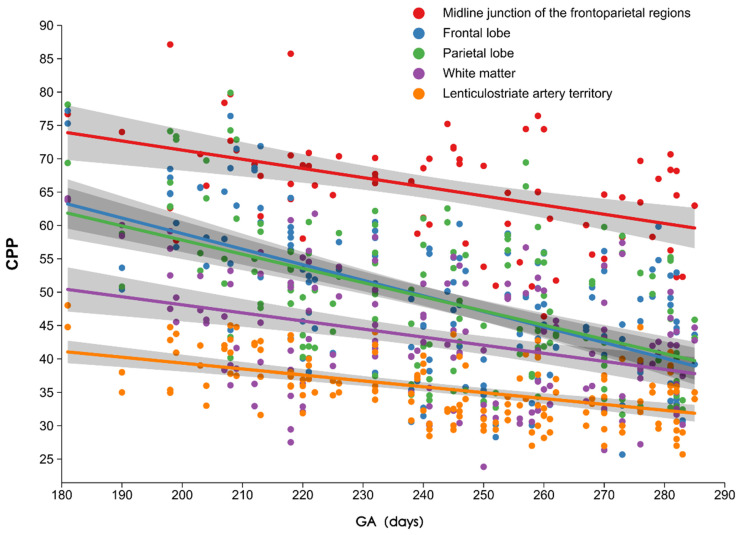
Relationship between the CPP value at full-term corrected gestational age and gestational age at birth.

Significant differences in CPP were detected among different brain regions within the three gestational age groups, and CPP also differed across gestational age groups. In the extremely preterm and very preterm groups, significant differences were detected in all brain regions, except the frontoparietal lobes. CPP values, ranked from highest to lowest, were observed in the midline, frontoparietal lobes, white matter, and lenticular arteries. In the moderate-to-late preterm and full-term infant groups, no significant difference in CPP was detected among the parietal lobe, frontal lobe, and white matter (Figure 4). Figure 4 shows the relationship between CPP during TEA and gestational age at birth. No significant difference was detected in bilateral CPP at the same site within each gestational age group.

### 3.4. Comparison of aEEG Scores Among the Three Groups

**Figure 5:**
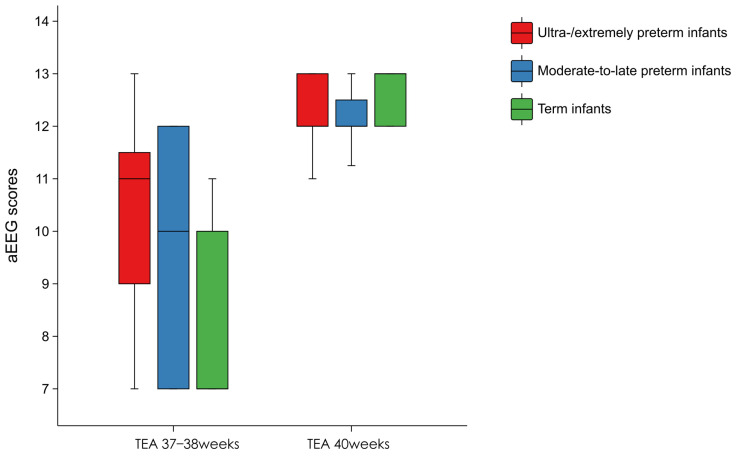
Relationship between the Burdjalov amplitude-integrated electroencephalogram (aEEG) scores at full-term corrected gestational age and the gestational age at birth.

Intergroup differences in the Burdjalov aEEG scores were significantly higher in preterm than in full-term infants when corrected to early term (37–38 weeks). At TEA 40 weeks ± 3 days, no significant intergroup difference was detected in the Burdjalov aEEG scores (Table 4 and Figure 5).

## 4. Discussion

This study systematically revealed the characteristics of cerebral microcirculation and EEG activity in preterm infants corrected to TEA through the combination of UMA and aEEG technology. Regardless of whether macroscopic monitoring of the ACA and PSV of the MCA or the microscopic monitoring provided by UMA, the cerebral perfusion index of extremely preterm infants and very preterm infants was significantly higher than that of infants with larger gestational age upon reaching the full-term corrected gestational age, consistent with previous reports [7,8,17]. This difference may be attributed to multiple factors. First, although premature infants have a longer latency of neural response, their cortical vascular coupling mechanism is intact, and longer extrauterine stimulation promotes synaptic formation and myelination, enhances brain functional activity, and thereby drives brain development [17]. Second, the most significant developmental growth in human cerebral blood flow occurs in the early postnatal period; with increasing age, the perfusion and metabolic levels of brain tissue increase simultaneously. These factors jointly shape the unique cerebral hemodynamic characteristics of premature infants during TEA [3,7,8,10].

There were regional differences in cerebral blood flow between preterm and full-term infants with TEA: the CPP value was highest in the midline area, followed by the frontal and parietal lobes and white matter, and lowest in the striatal artery-perfused area [18]. The midline area (a high-level center for the integration of sensorimotor functions) maintains a high physiological perfusion owing to its high metabolic demands [19]. The frontoparietal lobe CPP of extremely preterm and very preterm infants was higher than that in the white matter area. This difference was not significant in the moderate-to-late preterm and full-term infant groups; thus, the smaller the gestational age of preterm infants, the more pronounced the characteristics of cerebral cortical hyperperfusion at corrected full term. This may be attributable to continuous stimulation in the extrauterine environment, which increases local cerebral blood flow through enhanced neuronal activity, increased synaptic proliferation and myelination, and increased metabolic demands [3,7,20,21]. For the white matter area, the CPP value during TEA in extremely preterm and very preterm infants was higher than that in full-term infants; the smaller the gestational age, the higher the CPP value. Although this hyperperfusion phenomenon (specific to premature infants) possibly reflects accelerated compensatory maturation during development, this abnormal developmental trajectory may be closely related to impaired cognitive function [21]. Although UMA allows precise assessment of the perfusion characteristics of specific brain regions, the high perfusion state detected by UMA does not equate to fully mature brain function. Multimodal system evaluation is needed for a comprehensive judgment.

In recent years, aEEG and the Burdjalov scoring system have been verified as comprehensive evaluation indices of EEG maturity [22]. The Burdjalov score of preterm infants at 37–38 weeks of corrected gestational age was higher than that of full-term infants, with no significant differences observed at approximately 40 weeks of corrected gestational age. This leading role in EEG development and maturation only exists in the early corrected full-term period. Owing to the more abundant audiovisual and noxious stimuli in the postnatal environment, the influence of postnatal age on aEEG patterns exceeds the effect of gestational age growth [23]. Brain injury can affect EEG maturation. A low Burdjalov score at TEA is clearly correlated with delayed motor and cognitive development [24] and thus serves as an important biological predictor of neurodevelopmental outcomes [25].

Preterm infants without obvious brain development abnormalities have higher microcirculation perfusion in each brain region and higher Burdjalov scores than newborns of the same gestational age at full-term corrected gestational age. The smaller the gestational age, the more significant the difference. UMA combined with aEEG provides an accurate, real-time, simple, safe, and noninvasive multimodal monitoring method for determining the neurological development of premature infants.

This study has some limitations that should be acknowledged. First, as we focused on establishing normative perfusion patterns, neonates with major comorbidities were excluded; while methodologically justified, this limits the generalizability of our findings to high-risk preterm populations. Second, owing to the single-center study design, which included an exclusively Chinese cohort, our findings require validation across diverse ethnic groups to account for potential population stratification in cerebral hemodynamics. Third, the cross-sectional design precluded evaluation of long-term neurodevelopmental outcomes; thus, whether the observed high perfusion gradient in preterm infants confers beneficial or adverse effects in later life remains unclear. Fourth, while Bonferroni correction was applied for within-group comparisons, the unadjusted cross-outcome analyses and limited sample size (although adequate for detecting gestation-dependent differences) constrain the robustness of subgroup analyses, particularly for postnatal exposures or physiological variables; these relationships warrant further investigation.

In conclusion, by integrating UMA technology and aEEG, we elucidated cerebral microcirculation perfusion patterns and EEG activity characteristics of preterm infants of different gestational ages, corrected to TEA through multimodal monitoring. Premature infants exhibited a unique state of cerebral hyperperfusion during TEA. The smaller the gestational age, the more significant the perfusion difference, whereby extrauterine environmental stimuli potentially influence development trajectories of local hemodynamics by promoting neurometabolic activities. A simple hyperperfusion phenomenon may reflect compensatory rather than development-optimized blood flow remodeling or, alternatively, indicate immature autoregulation. aEEG analysis indicated that early EEG maturity was higher than that of full-term infants at TEA 37–38 weeks but tended to converge at 40 weeks. Combined UMA–aEEG evaluation could provide a reference for microcirculation perfusion in each brain region during neonatal brain development, especially in premature infants, and warrants wider clinical application, particularly for the neurological monitoring of high-risk preterm infants.

## Figures and Tables

**Table 1:** Burdjalov amplitude-integrated electroencephalogram scoring system.

Score	Continuity	Cycling	Amplitude of Lower Border	Bandwidth Span and Amplitude of Lower Border
0	Discontinuous	None	Severely depressed (<3 μV)	Very depressed: low span (≤15 μV) and low voltage (5 μV)
1	Somewhat continuous	Wave first appeared	Somewhat depressed (3–5 μV)	Very immature: high (>20 μV) or moderate (15–20 μV) span and low voltage (5 μV)
2	Continuous	Not definite, somewhat cycling	Elevated (>5 μV)	Immature: high span (>20 μV) and high voltage (>5 μV)
3		Definite cycling, but interrupted		Maturing: moderate span (15–20 μV) and high voltage (>5 μV)
4		Definite cycling, uninterrupted		Mature: low span (<15 μV) and high voltage (>5 μV)
5		Regular and mature cycling		

**Table 2:** Intergroup comparison of regional cerebral perfusion pressure.

	Ultra-/Extremely Preterm Infants, *n* = 23	Moderate-to-Late Preterm Infants, *n* = 27	Term Infants, *n* = 26	*p*-Value
Sex (females)	10 (43.478)	10 (37.037)	13 (50.000)	0.636
Gestational age (weeks)	30.3 [29.1–31.1]	34.9 [34.0–35.7] ^a^	39 [37.6–40.1] ^b,c^	<0.001
Birth weight (g)	1300 [1125–1500]	2160 [1910–2400] ^a^	3350 [3008–3510] ^b,c^	<0.001
SGA	2 (8.696)	3 (11.111)	1 (3.846)	0.609
Cesarean delivery	13 (56.522)	18 (66.667)	9 (34.615)	0.059
IVF	5 (21.739)	5 (18.519)	7 (26.923)	0.761
Gestational hypertension	5 (21.739)	4 (14.815)	2 (7.692)	0.377
Gestational diabetes mellitus	4 (17.391)	8 (29.630)	4 (15.385)	0.390
SLE with pregnancy	1 (4.348)	1 (3.704)	1 (3.846)	0.993
PMA (days)	276.3 ± 9.6	275.5 ± 8.2	278.0 ± 7.0	0.532
Age at examination (days)	66 [55–78]	28 [25–40.5] ^a^	5 [3–8] ^b,c^	<0.001
Weight at examination (g)	2607.4 ± 346.5	3268.9 ± 336.5 ^a^	3369.6 ± 281.6 ^b^	<0.001
HR (bpm)	140.5 ± 8.6	140.5 ± 9.5	135.9 ± 9.9	0.135
R (breaths pm)	40.7 ± 6.7	40.7 ± 5.2	39.2 ± 6.9	0.599
SBP (mmHg)	77.0 ± 5.8	79.1 ± 7.4	80.2 ± 6.1	0.436
DBP (mmHg)	43.4 ± 6.9	44.2 ± 6.7	46.0 ± 8.9	0.455
MAP (mmHg)	54.6 ± 6.0	55.8 ± 6.4	57.1 ± 6.9	0.410

Data are presented as mean ± SD, median [Q1, Q3], or n (%). ^a^ Ultra-preterm and extremely preterm infants differed significantly from moderate-to-late preterm infants; ^b^ Ultra-preterm and extremely preterm infants differed significantly from term infants; ^c^ Moderate-to-late preterm infants differed significantly from term infants. SLE, systemic lupus erythematosus; PMA, postmenstrual age; SGA, small-for-gestational age; IVF, in vitro fertilization; HR, heart rate; bpm, beats per minute; R, respiratory rate; SBP, systolic blood pressure; DBP, diastolic blood pressure; MAP, mean arterial pressure.

**Table 3:** Intergroup comparison of the regional cerebral perfusion pressure.

	Ultra-/Extremely Preterm Infants, *n* = 23	Moderate-to-Late Preterm Infants, *n* = 27	Term Infants, *n* = 26	*p*-Value
Midline junction of the frontoparietal regions	70.18 ± 7.66	64.87 ± 6.91 ^a^	61.75 ± 7.48 ^b^	0.001
Left frontal lobe	58.92 ± 10.28	45.31 ± 9.66 ^a^	43.81 ± 7.98 ^b^	<0.001
Right frontal lobe	57.28 ± 10.26	45.43 ± 9.28 ^a^	44.34 ± 8.82 ^b^	<0.001
Left parietal lobe	55.77 ± 11.13	46.30 ± 10.22 ^a^	43.82 ± 8.21 ^b^	<0.001
Right parietal lobe	56.30 ± 11.76	47.64 ± 10.13 ^a^	44.19 ± 8.26 ^b^	<0.001
Left white matter	46.90 ± 9.93	42.02 ± 8.36	40.45 ± 7.52 ^b^	0.030
Right white matter	46.95 ± 9.73	41.64 ± 8.86	40.54 ± 6.73 ^b^	0.024
Left lenticulostriate artery-perfused region	39.20 ± 3.75	34.93 ± 4.75 ^a^	34.06 ± 5.16 ^b^	<0.001
Right lenticulostriate artery-perfused region	38.98 ± 4.14	33.55 ± 3.62 ^a^	32.99 ± 4.58 ^b^	<0.001
ACA PSV (cm/s)	60.49 ± 9.61	49.64 ± 8.85 ^a^	41.96 ± 8.47 ^b,c^	<0.001
ACA EDV (cm/s)	15.02 ± 5.39	13.83 ± 2.81	11.85 ± 2.45 ^b^	0.013
ACA RI	0.75 ± 0.06	0.72 ± 0.07	0.71 ± 0.03 ^b^	0.029
MCA PSV (cm/s)	71.06 ± 10.33	62.04 ± 9.59 ^a^	53.18 ± 11.22 ^b,c^	<0.001
MCA EDV (cm/s)	19.59 ± 4.05	17.09 ± 5.08	15.99 ± 4.10 ^b^	0.020
MCA RI	0.72 ± 0.05	0.73 ± 0.06	0.70 ± 0.06	0.173

Data are presented as mean ± standard deviation. ^a^ Ultra-preterm and extremely preterm infants differed significantly from moderate-to-late preterm infants; ^b^ Ultra-preterm and extremely preterm differed significantly from term infants; ^c^ Moderate-to-late preterm infants differed significantly from term infants.

**Table 4:** Comparison of aEEG scores among the three infant groups.

Gestational Age (Days)	Ultra-/Extremely Preterm Infants, *n* = 23	Moderate-to-Late Preterm Infants, *n* = 27	Term Infants, *n* = 26	*p*-Value
259–266	11 (9, 11.5)	10 (7, 12)	7 (7, 10) ^b,c^	0.003
277–2	12 (12, 13)	12 (12, 12.5)]	13 (12, 13)	0.056

Data are presented as median [Q1, Q3]. ^b^ Ultra-preterm and extremely preterm differed significantly from term infants; ^c^ Moderate-to-late preterm infants differed significantly from term infants.

## Data Availability

All data generated or analyzed during this study are included in this article. Further enquiries can be directed to the corresponding author.

## Abbreviations

The following abbreviations are used in this manuscript:

ACA	Anterior cerebral artery
aEEG	Amplitude-integrated electroencephalogram
CDFI	Color Doppler flow imaging
CPP	Color pixel percentage (note: this abbreviation is context-specific and unrelated to cerebral perfusion pressure)
EDV	End-diastolic velocity
MCA	Middle cerebral artery
PSV	Peak systolic velocity
RI	Resistive index
TEA	Term-equivalent age
UMA	Ultra-micro angiography
